# Transcriptional regulation of drought response: a tortuous network of transcriptional factors

**DOI:** 10.3389/fpls.2015.00895

**Published:** 2015-10-29

**Authors:** Dhriti Singh, Ashverya Laxmi

**Affiliations:** National Institute of Plant Genome ResearchNew Delhi, India

**Keywords:** ABA, drought, regulons, cross-talk, transcription factors

## Abstract

Drought is one of the leading factors responsible for the reduction in crop yield worldwide. Due to climate change, in future, more areas are going to be affected by drought and for prolonged periods. Therefore, understanding the mechanisms underlying the drought response is one of the major scientific concerns for improving crop yield. Plants deploy diverse strategies and mechanisms to respond and tolerate drought stress. Expression of numerous genes is modulated in different plants under drought stress that help them to optimize their growth and development. Plant hormone abscisic acid (ABA) plays a major role in plant response and tolerance by regulating the expression of many genes under drought stress. Transcription factors being the major regulator of gene expression play a crucial role in stress response. ABA regulates the expression of most of the target genes through ABA-responsive element (ABRE) binding protein/ABRE binding factor (AREB/ABF) transcription factors. Genes regulated by AREB/ABFs constitute a regulon termed as AREB/ABF regulon. In addition to this, drought responsive genes are also regulated by ABA-independent mechanisms. In ABA-independent regulation, dehydration-responsive element binding protein (DREB), NAM, ATAF, and CUC regulons play an important role by regulating many drought-responsive genes. Apart from these major regulons, MYB/MYC, WRKY, and nuclear factor-Y (NF-Y) transcription factors are also involved in drought response and tolerance. Our understanding about transcriptional regulation of drought is still evolving. Recent reports have suggested the existence of crosstalk between different transcription factors operating under drought stress. In this article, we have reviewed various regulons working under drought stress and their crosstalk with each other.

## Introduction

Plants being sessile organisms frequently encounter a wide range of unfavorable conditions during their life cycle. These conditions have deleterious effects on their physiology leading to reduced growth and development. Such adverse conditions along with some other factors play a crucial role in determining the yield and geographical distribution of plants. These different unfavorable conditions are generally termed as stress.

Plants face both abiotic as well as biotic stresses during their life cycle. Various climatic factors such as extreme temperature, drought, salinity, and chemical contamination of soil fall in the category of abiotic stresses. However, stresses caused by various pathogens and other biological agents are grouped into biotic stress. Both kinds of stresses have detrimental effects on plants. Especially, abiotic stresses alter various cellular processes such as photosynthesis, growth, carbon partitioning, carbohydrate and lipid metabolism, protein synthesis, gene expression, and osmotic homeostasis. Thus, to survive under stress conditions, plants have evolved a wide range of mechanisms to avoid or tolerate these stresses.

Drought is an imperative factor limiting the crop productivity across the globe (Bray et al., 2000). It can be characterized by below normal precipitation for a certain period of months to year and drying winds leading to reduced soil water available to plants. In addition to this, it is generally accompanied with high temperature. In recent years, frequency of drought stress has increased due to irregular rain fall. Almost every year drought occurs in some part of the earth reducing the crop yield. The condition is going to be worse in coming years due to global warming responsible for increasing desertification. On the other hand, world population is anticipated to reach 9 billion by 2050 (http://www.fao.org/wsfs/world-summit/en). Taking into consideration the increase in population, it is important to increase crop yield. Therefore, it is important to understand the mechanism of drought stress tolerance in plants in order to improve crop productivity under stress conditions.

Understanding the mechanism underlying drought stress tolerance has been an active area of research. Till date, drought stress responses have been studied in various plants; including crops, vegetables, trees as well as horticulture plants. Plants respond to environmental stresses at various levels such as cellular responses, metabolic changes, molecular adaptations as well as epigenetic regulation (Krasensky and Jonak, 2012). Although, plant response to drought has been analyzed at all these levels but in past few years focus has shifted toward the molecular mechanism. Drought stress affects the expression of many genes. Most of the molecular studies have been done using *Arabidopsis thaliana* as a model plant (Ingram and Bartels, 1996; Shinozaki and Yamaguchi-Shinozaki, 2000). Genome sequence of *Arabidopsis* has provided valuable information pertaining genes, gene families, *cis* elements and other related factors; resulting in rapid progress regarding molecular responses of plants to drought (Hirayama and Shinozaki, 2010). Later on, in addition to genomics, incorporation of advanced omics approaches such as transcriptomics, proteomics and metabolomics have increased our knowledge in this area (Hirayama and Shinozaki, 2010).

During drought and other osmotic stresses, the phytohormone ABA (abscisic acid) plays a pivotal role in plant adaptation. Effect of ABA on plant response to stress has been extensively researched. ABA is accumulated under drought stress condition due to induction of ABA biosynthetic genes (Iuchi et al., 2001). ABA regulates the expression of many genes leading to some important physiological as well as biochemical changes that help plant to survive under stress (Umezawa et al., 2010). Molecular and genomic analyses have revealed the existence of ABA-independent signal transduction pathway in conjunction to the ABA-dependent signal transduction pathway during drought stress (Yamaguchi-Shinozaki and Shinozaki, 2006).

## Transcriptional Regulatory Network

Plants respond to various environmental stresses including drought through changes ranging from physiological to molecular level. These changes help plants to optimize their growth and stress resistance. Drought stress changes the expression of many genes that are thought to play an important role in stress response and tolerance. Many of these genes have been identified and characterized (Yamaguchi-Shinozaki and Shinozaki, 2006; Todaka et al., 2015). Microarray analyses by various groups have revealed thousands of genes that are upregulated and downregulated in response to drought stress. A significant number of drought-inducible genes are also induced by high salinity, suggesting a cross-talk between drought and salt stress. Comparatively lesser number of drought-inducible genes are induced by cold stress (Yamaguchi-Shinozaki and Shinozaki, 2006). There is a very small overlap of only 27 genes that were found to be commonly induced in microarray studies (Bray, 2004). This lack of commonality may be attributed to the fact that different sets of probes were used during these microarray experiments and variations in conditions of plant growth and stress. Recently, 17 microarray experiments of *Arabidopsis*, rice, wheat, and barley were compared using a novel **C**ross-**S**pecies meta-**A**nalysis of progressive **Drought** stress at the reproductive stage (**CSA:Drought**); and 225 differentially expressed genes were identified that were shared across studies and taxa (Shaar-Moshe et al., 2015).

Stress inducible genes in *Arabidopsis* can be classified into two categories: functional and regulatory genes (Yamaguchi-Shinozaki and Shinozaki, 2006). Genes encoding proteins required for cellular stress tolerance fall into the former category, for example, LEA (late embryogenesis abundant) proteins, molecular chaperones, reactive oxygen species detoxifying enzymes, and sugars or proline biosynthetic enzymes. Whereas, genes encoding proteins that are involved in signal transduction and gene expression come under the latter category, such as protein kinases, components of ABA signaling, enzymes for lipid signaling, and various transcription factors (Yamaguchi-Shinozaki and Shinozaki, 2006).

As stated above, plant hormone ABA plays an important role in response to water deficit including regulation of transcriptional network (Yamaguchi-Shinozaki and Shinozaki, 2006). A large number of genes that are induced by water deficit are also highly induced by exogenous application of ABA. Conversely, there are several genes that are induced by water deficit but are not affected by exogenous ABA. These findings suggested that transcriptional response to water deficit is regulated by both ABA-dependent and ABA-independent signal transduction pathways (Yamaguchi-Shinozaki and Shinozaki, 2006).

Transcription factors are regulatory proteins that can modulate expression of a specific set of genes through binding to their promoter. They play an important role in converting the stress-induced signals to cellular responses. A single transcription factor can modulate the expression of a number of genes. A collection of genes under regulation of the same regulatory protein is called regulons. Plants activate many regulons under drought and other stresses to optimize plant growth, some of them have been well determined in *Arabidopsis* (Nakashima et al., 2009). Both ABA-dependent and ABA-independent pathways regulate the transcriptional response by affecting one or more regulons active under drought stress (Nakashima et al., 2009). In the following section, we will discuss in brief about water deficit induced regulons and pathways affecting them.

### AREB/ABF Regulon

**FIGURE 1:**
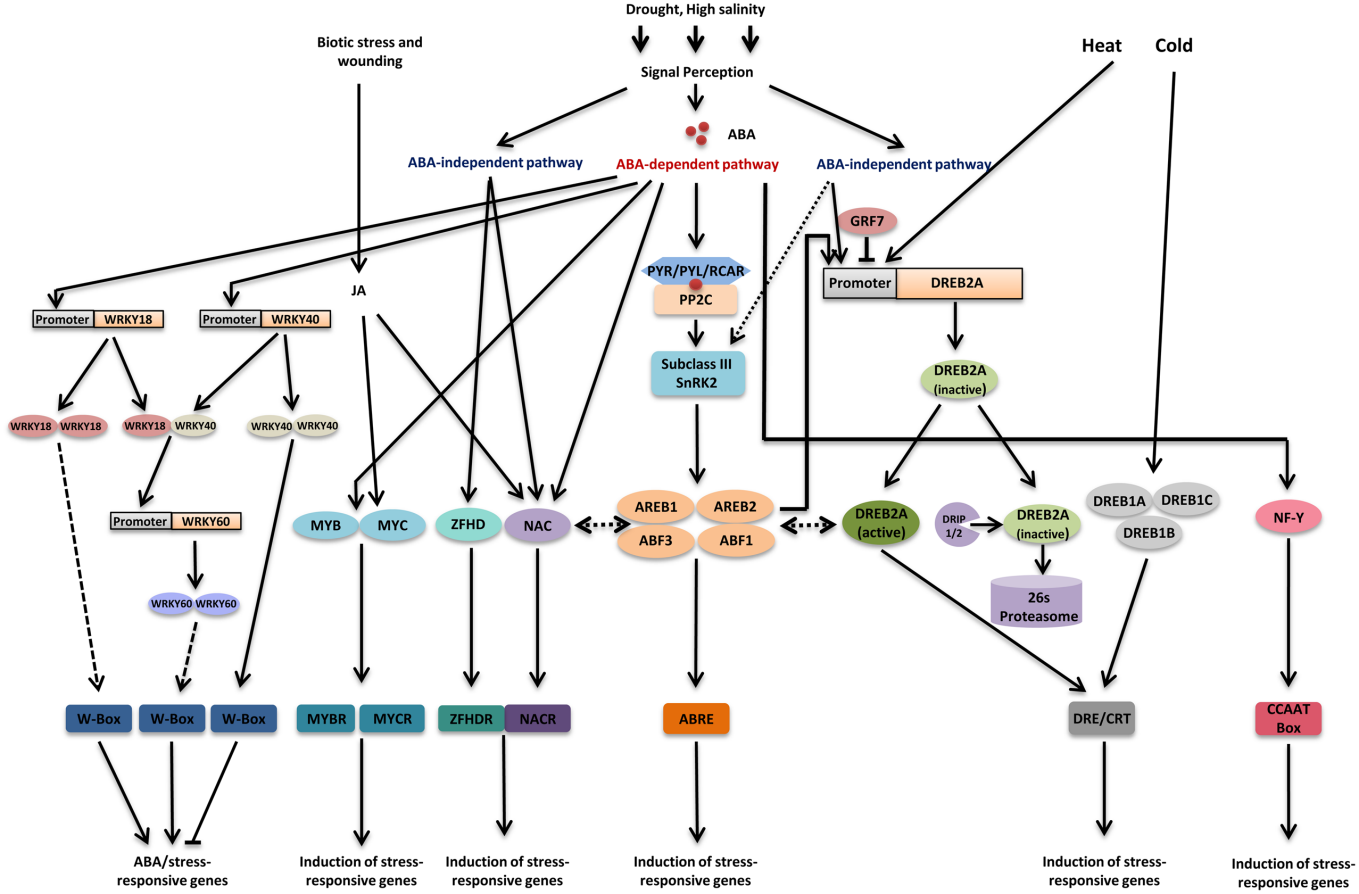
**Major transcriptional regulatory networks of transcription factors involved in drought stress.** Drought signal perception leads to activation of both abscisic acid (ABA)-dependent and ABA-independent pathways. In ABA-dependent pathway, accumulation of ABA leads to activation of sub class III SnRK2s through PYR/PYL/RCAR-PP2C receptor complex. Four transcription factors ABA-responsive element (ABRE) binding protein 1 (AREB1), AREB2, ABRE binding factor 3 (ABF3), and ABF1 are mainly phosphorylated through sub class III SnRK2s under drought stress and regulate most of the downstream genes by binding to the ABRE *cis*-element present in their promoter region. In addition to this, ABA also modulates the activity of MYB/MYCs, NACs, WRKYs, and NF-Y transcription factors. MYC2 and NAC proteins are also involved in JA signaling. ABA signal perception leads to induction of WRKY18 and WRKY40 and their product could bind to W-box present in WRKY60 and thereby induce it. DREB2A plays a pivotal role in ABA-independent gene expression regulation under drought stress. *DREB2A* expression is regulated by GRF7 under unstressed condition. Additionally, DRIPs regulate levels of DREB2A protein under unstressed condition. DREB2A also participate in gene expression regulation under heat stress. Three DREB1 proteins, DREB1A, DREB1B, and DREB1C are the key factors regulating gene expression under cold stress. Some NAC transcription factors also regulate gene expression in ABA-independent manner. Recently, it has been reported that AREB/ABFs can induce DREB2A and AREB/ABFs interact with DREB2A, based on these observations, ABA-dependent and ABA-independent pathways are thought to crosstalk under drought stress. Apart from this, some recent reports suggest a possible interaction between NACs and AREB/ABFs. Transcription factors and DNA-binding proteins are shown in colored ellipses. Dashed lines indicate possible although unconfirmed routes.

Abscisic acid-responsive element binding protein (AREB) /ABF (ABRE binding factor) regulon function in ABA-dependent regulation of gene expression under drought stress (Nakashima et al., 2009; Yoshida et al., 2015), (**Figure 1**). Many genes that are affected by water deficit also respond to the exogenous application of ABA (Nakashima et al., 2009). Promoter analysis revealed that most of these ABA-responsive genes are regulated by ABRE (ABA-responsive element) in their promoter region. ABRE is a conserved, 8 bp long *cis* element (PyACGTGG/TC) with a core ACGT sequence (Nakashima et al., 2009; Fujita et al., 2011). A single copy of an ABRE is not sufficient to induce ABA-responsive gene expression. To function as an active *cis*-acting element, ABRE requires in proximity other copies of ABRE or another specific *cis*-acting element, which is termed as the coupling element. Certain sequences have been shown to function as coupling elements such as, CE1 (coupling element1) and CE3 (coupling element3); and DRE (dehydration-responsive element)/CRT (C-repeat) *cis* element (major *cis* element in ABA-independent pathway; Shen et al., 1996; Narusaka et al., 2003). CEs are usually GC-rich sequence and they are similar to ABRE (Yamaguchi-Shinozaki and Shinozaki, 2006).

Abscisic acid-responsive element binding protein or ABF transcription factors were found to bind to ABRE element in yeast one-hybrid screening. These are major transcription factors that bind to ABRE and regulate ABA-responsive gene expression (Choi et al., 2000; Uno et al., 2000), (**Figure 1**). AREB/ABF is a subfamily of the basic leucine zipper (bZIP) family that consists of 9 members in *Arabidopsis.* All AREB/ABF transcription factors contain four conserved domains in addition to bZIP domain (Fujita et al., 2011, 2013). In *Arabidopsis, AREB1/ABF2, AREB2/ABF4, ABF1*, and *ABF3* are expressed mainly in the vegetative tissues in response to ABA and osmotic stress (Fujita et al., 2011). In contrast, some other members are expressed during seed maturation such as *Arabidopsis ABI5, AREB3, DPBF2*, and *EEL* (Finkelstein and Lynch, 2000; Lopez-Molina and Chua, 2000; Bensmihen et al., 2002). Transgenic plants overexpressing *AREB1/ABF2, AREB2/ABF4*, or *ABF3* exhibit enhanced drought tolerance and increased ABA sensitivity (Kang et al., 2002; Fujita et al., 2005). Triple AREB/ABF mutant *areb1 areb2 abf3* exhibits reduced tolerance to drought and decreased sensitivity to exogenous ABA compared to that of wild-type, single mutants, or double mutants (Yoshida et al., 2010). Transcriptome analysis of the triple mutant under osmotic stress conditions showed reduced levels of many osmotic stress-inducible genes (Yoshida et al., 2010). Recently, ABF1 has also been reported to play an important role in ABA-mediated gene expression under drought stress. Although, it is expressed in lesser quantity as compared to those three AREB/ABFs, the *areb1 areb2 abf3 abf1* quadruple mutant plants show an increase in drought sensitivity and decreased ABA sensitivity in comparison to *areb1 areb2 abf3* mutant. In quadruple mutant many dehydration-inducible genes including LEA protein genes and transcription factors show reduced expression (Yoshida et al., 2015). Both triple mutants, as well as quadruple mutants, exhibited decreased inflorescence heights as compared to wild-type; as well as delayed bolting. Except for this, all of them showed phenotypes similar to wild-type (Yoshida et al., 2010, 2015). Thus, these four AREB/ABFs have been shown to be central transcription factors that cooperatively function in ABA-dependent transcriptional activation through their ABREs under these abiotic stress conditions (**Figure 1**).

Abscisic acid-responsive element binding protein/ABF trans cription factors are fully activated only after phosphory lation of their conserved regions (Fujita et al., 2011). The phosphorylation is catalyzed by serine/threonine kinase SnRK2s (SNF1-related protein kinase) that are induced by ABA. The importance of phosphorylation was suggested by the observation that overexpression of *AREB1/ABF2* activated downstream gene expression only when phosphorylated active form of this gene was used (Furihata et al., 2006). Thus, under water deficit, cellular ABA concentration is increased that is recognized by the ABA receptors PYR/PYL/RCARs (pyrabactin resistance/pyrabactin resistance1- like/regulatory component of ABA receptors) leading to the inhibition of phosphatase activity of PP2C (protein phosphatase 2C). PP2C is a negative regulator of ABA signaling which dephosphorylates and thereby inactivates subclass III SnRK2s. Subsequently, released subclass III SnRK2s get accumulated in the cell and phosphorylate AREB/ABF thereby inducing expression of AREB/ABF regulon genes (reviewed in Cutler et al., 2010; Umezawa et al., 2010), (**Figure 1**). There are 10 members of SnRK2 family in *Arabidopsis* that are divided into three groups including three members in subclass III SnRK2s, SRK2D/SnRK2.2, SRK2E/OST1/SnRK2.6, and SRK2I/SnRK2.3 (Umezawa et al., 2010). Out of these 10 SnRK2s, nine are activated by osmotic stress. However, subclass III SnRK2s are also strongly induced by ABA and mediate most of the ABA responses (Yoshida et al., 2014). Subclass III SnRK2s have been shown to phosphorylate AREB/ABFs *in vitro* as well as co-localize and interact with them in plant cell nuclei (Furihata et al., 2006; Fujii et al., 2007; Fujita et al., 2009; Yoshida et al., 2010). Studies involving triple mutant *srk2d/e/i* showed that expression of most of the AREB1/ABF2, AREB2/ABF4, and ABF3 regulated genes are highly reduced and ABA-dependent phosphorylation of AREB/ABFs is completely abolished (Fujii and Zhu, 2009; Fujita et al., 2009). All these results indicate that subclass III SnRK2s regulate ABA-responsive gene expression under drought stress by phosphorylating AREB/ABFs.

### DREB1/CBF and DREB2 Regulons

Dehydration-responsive element binding protein 1 (DREB1)/CBF (C-repeat binding factor) and DREB2 regulons function in ABA-independent regulation of gene expression under drought stress (Nakashima et al., 2009), (**Figure 1**). In *Arabidopsis*, the *RD29A/COR78/LTI78* gene is induced by drought and cold. This gene has been found to be ABA-inducible but at the same time it can be induced by drought and cold stress in mutants defective in ABA biosynthesis and signaling (Yamaguchi-Shinozaki and Shinozaki, 2006). The analysis of its promoter, together with expression studies suggested that the dehydration inducibility of this gene is regulated by both ABA-independent and ABA-dependent pathways through different *cis*-acting elements (Yamaguchi-Shinozaki and Shinozaki, 2006).

The promoter of *RD29A* gene contains DRE/CRT *cis* element in addition to ABRE (Yamaguchi-Shinozaki and Shinozaki, 1994, 2005). DRE element is responsible for ABA-independent induction of many genes in response to osmotic and cold stress in many plants including *Arabidopsis* (Yamaguchi-Shinozaki and Shinozaki, 2005). DRE is a conserved 9 bp long (TACCGACAT) *cis* element. Unlike ABRE, a single copy of DRE is sufficient to induce genes under osmotic and cold stress (Yamaguchi-Shinozaki and Shinozaki, 1994). Similar *cis*-acting elements, named C-repeat (CRT) and low-temperature responsive element (LTRE), were identified in low-temperature-inducible genes. These sequences share a common core sequence, A/GCCGAC, which is referred to as the DRE/CRT core sequence (Baker et al., 1994; Jiang et al., 1996; Thomashow, 1999). DRE/CRTs are found in promoters of many stress-inducible genes.

Dehydration-responsive element binding protein 1/CBF and DREB2 transcription factors recognize DRE/CRT and activate downstream genes (**Figure 1**). Both DREB1/CBF and DREB2 belong to the plant-specific AP2 (APETALA2)/ ERF (ethylene-responsive element-binding factor) family having AP2/ERF DNA-binding motif. There are 145 members in AP2/ERF transcription factor family in *Arabidopsis* (Sakuma et al., 2002). DREB transcription factors constitute a subfamily of AP2/ERF family. *Arabidopsis* has six and eight *DREB1/CBF*-type and *DREB2*-type genes, respectively. Among them, three DREB1/CBF-type transcription factors, DREB1A/CBF3, DREB1B/CBF1, and DREB1C/CBF2 are rapidly induced by low temperature and act as major transcription factors that activate gene transcription through DRE/CRT in response to cold stress (Nakashima et al., 2009), (**Figure 1**). Transgenic plants overexpressing *DREB1* show enhanced tolerance to cold and accumulate osmoprotectants such as proline and various sugars (Gilmour et al., 2000). These transgenic plants also exhibit “dwarf” phenotype and pronounced prostrate growth habits. They have shorter petiole in comparison to wild-type plants with leaves having bluish-green tint. Additionally, plants overexpressing *DREB1* show delayed bolting and flowering as well as lower yield in comparison to wild-type plants (Gilmour et al., 2000).

In contrast to DREB1, two DREB2-type transcription factors, DREB2A and DREB2B, are highly induced in response to osmotic stress conditions and are considered to be involved in DRE-mediated gene transcription in response to water deficit (Liu et al., 1998), (**Figure 1**). Later, it was found that DREB2A plays a pivotal role in gene expression regulation under salt stress whereas DREB2B regulates gene expression in response to drought stress (Nakashima et al., 2009). However, weak induction of several *DREB1* genes such as *DREB1D/CBF4, DREB1E/DDF2*, and *DREB1F/DDF1* under dehydration stress suggests that DREB1/CBF and DREB2 regulons interact with each other (Haake et al., 2002; Sakuma et al., 2002; Magome et al., 2004).

Although DREB2A and DREB1A were isolated together (Liu et al., 1998), later, it was discovered that both of them have slight difference in their downstream genes (Maruyama et al., 2009). Microarray analysis has suggested that products of most of the genes downstream to DREAB1A and DREB2A have similar putative functions, but carbohydrate metabolism genes have different expression pattern in DREB1A and DREB2A transgenic plants (Maruyama et al., 2009). Plants overexpressing *DREB1A* exhibit changes in expression of genes responsible for starch degradation, sucrose metabolism and sugar alcohol synthesis similar to that observed in dehydration and cold stress. These changes lead to accumulation of many kinds of sugar and sugar alcohols in plants that might be responsible for enhanced dehydration and cold stress tolerance. In contrast plants overexpressing *DREB2A-CA* (constitutively active form of *DREB2A*) do not exhibit the increase in these metabolites level (Maruyama et al., 2009). The reason for this might be the difference in their DNA-binding specificity (Nakashima et al., 2009). DREB1A has a high affinity to A/GCCGACNT sequences, whereas DREB2A preferentially binds ACCGAC motifs (Sakuma et al., 2006a) resulting in slight variation in target genes.

Although DREB2A regulates the expression of many genes involved in stress response and tolerance, it causes growth retardation and reduced reproduction rate in plants, therefore, its expression is tightly regulated (Yoshida et al., 2014). *DREB2A* expression is negatively regulated by GRF7 (growth-regulating factor7). GRF proteins are a family of putative transcription factors that consist of nine members in *Arabidopsis* (Kim et al., 2003). Among these nine members, GRF7 inhibits expression of *DREB2A* under normal conditions by binding to its short promoter region (**Figure 1**). Both knockdown and knockout mutants of *GRF7* exhibit enhanced DREB2A expression under non-stressed condition (Kim et al., 2012a). These plants also exhibit enhanced salinity tolerance and retarded growth. Microarray analysis revealed that a large number of osmotic stress-responsive genes were upregulated in *grf7* knockout mutants under non-stressed condition. These shreds of evidence suggest that GRF7 regulates a large number of osmotic stress responsive genes by regulating *DREB2A* expression (Kim et al., 2012a).

In addition to transcriptional regulation, DREB2A is also regulated at post-transcriptional level. Transgenic plant overexpressing DREB1/CBF under *Arabidopsis* stress-responsive RD29A promoter showed strong tolerance to stresses mainly against cold (Kasuga et al., 1999). However, transgenic plant overexpressing *DREB2A* did not exhibit any significant phenotypic change (Liu et al., 1998). Domain analysis revealed that central region of DREB2A protein has a negative regulatory domain (NRD), and removal of the NRD from DREB2A converts the protein into its constitutively active form (DREB2A-CA). The DREB2A-CA proteins are more stable in the nucleus than the wild-type protein. In contrast to wild-type *DREB2A*, overexpression of *DREB2A-CA* shows enhanced drought tolerance as well as a slight improvement in cold stress tolerance and upregulation of many stress-inducible genes (Sakuma et al., 2006a). Transgenic plants overexpressing *DREB2A-CA* exhibited growth retardation in comparison to wild-type as well as rounded, slightly darker leaves with short petiole. Additionally, extent of retardation and these mentioned phenotypes was in correlation to the expression of transgene (Sakuma et al., 2006a). All these reports suggest that stability control is a posttranslational regulatory mechanism of DREB2A.

Furthermore, DRIP1 (DREB2A-interacting protein 1), a ubiquitin E3 ligase is thought to degrade the leaky expression under normal conditions (Qin et al., 2008). DRIP1 harbors a C3HC4-type RING domain and has been found to interact with DREB2A in yeast two-hybrid screening. Transgenic *Arabidopsis* plants overexpressing *DRIP1* exhibit delayed expression of DREB2A-regulated drought responsive genes, however, double knockout mutants of *DRIP1* and its homolog *DRIP2* exhibit increased expression of these genes. DRIP1 and DRIP2 function as E3 ubiquitin ligase and target DREB2A to 26S proteasome proteolysis and thereby negatively regulate the expression of drought-responsive genes (Qin et al., 2008), (**Figure 1**). Recently, it has been shown that stress signal play important role in stabilization of DREB2A (Morimoto et al., 2013). In *drip1drip2* mutant, DREB2A protein levels were induced rapidly under heat stress suggesting their involvement in DREB2A degradation but the protein levels reduced in the mutant by prolonged heat stress (Morimoto et al., 2013). It has also been shown that stabilization of DREB2A is important but not sufficient for induction of downstream genes (Morimoto et al., 2013). All these results suggest that apart from DRIPs some other factors might be involved in degradation and activation of DREB2A.

Recently, transcription factor ERF53 (ethylene response factor53) and two homologous C3HC4-type RING E3 ligases, RGLG2 (RING domain ligase2) and RGLG1 has been identified that also function similar to DREB2A and DRIPs and regulate drought stress-responsive genes (Cheng et al., 2012). ERF53 is an AP2/ERF transcription factor that belongs to the non-DREB2 subfamily. *AtERF53* expression increase significantly under drought and high salinity but it exhibit mild induction to exogenous ABA (Cheng et al., 2012; Hsieh et al., 2013). Overexpression of *AtERF53* induces unstable drought tolerance. AtERF53 has been found to interact with RGLG2 and RGLG1, both of which act as E3 ubiquitin ligase and target it to proteasomal degradation. AtERF53-GFP fusion protein accumulates more stably in double mutant *rglg1 rglg2* leading to enhanced drought stress tolerance. All these reports suggest that AtERF53 and RGLGs function in combination to regulate osmotic stress-responsive genes (Cheng et al., 2012; Hsieh et al., 2013).

Dehydration-responsive element binding protein 2A regulon also operates under heat shock stress (**Figure 1**). Overexpression studies of *DREB2A-CA* showed improved thermotolerance by inducing expression of heat shock related genes (Sakuma et al., 2006b; Mizoi et al., 2012). Similarly, transgenic plants overexpressing *DREB2C* showed increased expression of heat shock stress-inducible genes, leading to thermotolerance (Lim et al., 2007). Therefore, it is clear that DRE/CRT is involved in gene expression not only during dehydration but also under conditions of low and high temperature.

### NAC Regulon

NAM, ATAF, and CUC (NAC) are plant-specific transcription factors that constitute one of the largest families of plant transcription factors. There are more than a hundred members in *Arabidopsis* and rice that have been classified in 10 groups based on their phylogenetic relationship (Jensen et al., 2010). NAC transcription factors have a highly conserved N-terminal DNA-binding domain and variable C-terminal region this C-terminal region is thought to play a crucial role in determination of their target genes (Nuruzzaman et al., 2013). NAC transcription factors are involved in various developmental processes from shoot meristem development to auxin signaling (Olsen et al., 2005). There are reports demonstrating their involvement in abiotic and biotic stress signaling (reviewed in Olsen et al., 2005; Nakashima et al., 2012; Nuruzzaman et al., 2013). NAC transcription factors involved in stress response and tolerance have been classified in stress-responsive NAC (SNAC) group.

The *ERD1* (*early response to dehydration1*) gene encodes a ClpA homolog of *Arabidopsis* (Nakashima et al., 1997). ERD1 was found to be induced by osmotic stress such as dehydration and salt stress as well as during senescence; however, exogenous ABA application could not strongly induce it (Nakashima et al., 1997). Two different *cis*-acting elements, a MYC-like sequence (CATGTG) and a 14-bp *rps1* site 1-like sequence are necessary for dehydration induced expression of *ERD1* (Simpson et al., 2003). Three NAC transcription factors; ANAC019, ANAC055, and ANAC072/RD26 were reported to bind MYC-like sequence present in the promoter of ERD1 (Tran et al., 2004). These three proteins are included in the SNAC group of NAC transcription factors (Nakashima et al., 2012). Detailed DNA binding assay of these NAC transcription factors determined NACRS (NAC recognition sequence) ANNNNNTCNNNNNNNACACGCATGT, containing CATGT and harboring CACG as the core DNA-binding site (Tran et al., 2004), (**Figure 1**). These NAC genes were found to be expressed within 1–2 h of ABA treatment suggesting that they are induced through ABA-independent pathway under drought stress (Tran et al., 2004). Transgenic plants overexpressing *ANAC019* and *ANAC072* showed phenotype and the time course of growth similar to that of vector control (Tran et al., 2004). In contrast, plants overexpressing *ANAC055* exhibited growth rate similar to that of vector control until they reached rosette stage; after this point, plants in which the expression of transgene was at the medium level, showed a little delay in bolting as compared to vector control whereas; plants in which the transgene was overexpressed at high level remained at rosette stage for an additional few days before first bolting (Tran et al., 2004). Transgenic plant overexpressing *ANAC019, ANAC055*, or *ANAC072/RD26* induced expression of many stress-inducible genes but failed to up-regulate *ERD1* (Fujita et al., 2004; Tran et al., 2004).

Subsequently, a zinc-finger homeodomain (ZFHD) transcription factor, ZFHD1, was identified as a transcriptional activator that recognizes the 14-bp rps1 site1-like sequence (CACTAAATTGTCAC) and this sequence was termed as ZFHDR (zinc finger homeodomain recognition sequence; Tran et al., 2007), (**Figure 1**). Expression of the *ERD1* gene was induced only when both the NAC and ZFHD proteins were overproduced simultaneously in a transgenic plant. Thus, these transcription factors cooperatively activate the transcription of the *ERD1* gene (Tran et al., 2007). Studies suggest that in addition to the role of ZFHD1 in cooperation of NAC transcription factor it can also function as transcriptional activator alone (Tran et al., 2007).

Overexpression of several stress-responsive NAC factors in *Arabidopsis* and rice has imparted drought tolerance in transgenic plants. For example, transgenic plants overexpressing *ANAC072/RD26* exhibited enhanced drought tolerance as well as increased sensitivity to ABA (Fujita et al., 2004; Tran et al., 2004). Microarray analysis of *ANAC072/RD26* overexpressing plants showed upregulation of stress-inducible genes and ABA-responsive genes suggesting that RD26 is involved in regulation of drought-responsive genes in ABA-dependent manner (Fujita et al., 2004). Similarly, overexpression of another NAC factor *ATAF1* resulted in improved drought tolerance (Wu et al., 2009). Recently, NAC genes such as *ANAC096* and *ANAC016* are associated with drought response and tolerance (Xu et al., 2013; Sakuraba et al., 2015). In case of rice, overexpression of *NAC* genes such as *SNAC1, OsNAC6/SNAC2, OsNAC5*, and *OsNAC10* improved drought tolerance (reviewed in Nakashima et al., 2014). NTLs (NAC with transmembrane motif 1-like) such as NTL4 and NTL6 are also involved in drought stress. Transgenic plants overexpressing *NTL6* and *ntl4* null mutants exhibit drought tolerance, suggesting that these two work antagonistic to each other during drought stress (Lee and Park, 2012; Kim et al., 2012b).

Several stress-inducible NAC genes are also induced by jasmonates and/or during senescence in *Arabidopsis* and rice (**Figure 1**). Thus, these stress-responsive NAC transcription factors not only function in the transcriptional response to abiotic stress conditions, including water stress, but are likely involved in the cross talk between abiotic and biotic stress responses (Nakashima et al., 2012).

### Other Transcriptional Pathways Involved in Water Stress Responses

In addition to the above-mentioned pathways of *cis*-acting elements and transcription factors, many other transcriptional pathways function in water stress responses. The *Arabidopsis RD22* gene is inducible by drought stress in ABA-dependent manner (Yamaguchi-Shinozaki and Shinozaki, 1993). Although it is induced by ABA, it does not have ABRE *cis*-element in its promoter region; in spite of that its expression is regulated by two *cis*-acting elements, MYC and MYB recognition elements (Abe et al., 1997). A MYC like transcription factor, MYC2, and a MYB transcription factor, MYB2 bind to these *cis*-acting elements and cooperatively activate the transcription of this gene (Abe et al., 1997, 2003), (**Figure 1**). Transgenic plants overexpressing *AtMYC2* and *AtMYB2* exhibit higher ABA sensitivity as well as osmotic tolerance (Abe et al., 2003). Transgenic plants overexpressing *AtMYC2* show morphology similar to wild-type. However, overexpression of *AtMYB2* causes growth retardation in transgenic plants growing on soil. Similarly, overexpression of both transcription factors causes severe growth retardation in plants growing on soil (Abe et al., 2003). Although, *AtMYC2* overexpressing plants have the morphology similar to wild-type, they have characteristically irregular shaped leaf epidermal cells. In contrast, plants overexpressing *AtMYB2* and both transcription factors have leaf epidermal as well as parenchymal cells similar to wild-type but smaller in shape (Abe et al., 2003). Microarray analysis has suggested that their target genes include many ABA-inducible genes. Conversely, a mutation in MYC2 decreased the expression of target genes, including *RD22* (Abe et al., 2003). All these results suggest that in addition to ABRE mediated gene regulation, MYB and MYC transcription factors regulate gene expression in response to ABA under drought stress (**Figure 1**).

MYB transcription factors are one of the largest transcription factor families in plants that have characteristic MYB domain in their DNA binding region (Lindemose et al., 2013). Analysis of transcriptome data present in GENEVESTIGATOR database showed that 51% of *Arabidopsis MYB* genes are upregulated and 41% are downregulated under drought stress (Baldoni et al., 2015). Furthermore, many MYB genes have been found to be involved in drought stress responses (reviewed in Baldoni et al., 2015).

MYC transcription factors belong to bHLH (basic-helix-loop-helix) transcription factor family of plants that have a characteristic bHLH domain (Kazan and Manners, 2013). Guard cell transcriptome analysis showed that ABA-responsive genes having MYC-binding motifs in the promoter region are present in large number in these cells (Wang et al., 2011). MYC2 protein has emerged as a master player in jasmonic acid signaling as well as cross-talk between jasmonic acid and ABA signaling (Kazan and Manners, 2013).

The involvement of WRKY transcription factors in drought stress has been reported recently. WRKY transcription factors constitute a family that has one or two WRKY domains which is involved in DNA binding. WRKY transcription factors bind to a conserved sequence named as W box and regulate gene expression (Rushton et al., 2010), (**Figure 1**). WRKY transcription factors are involved in various plant processes including biotic stress responses (Ulker and Somssich, 2004; Rushton et al., 2010). Recently, they have been reported to be involved in abiotic stress responses (Rushton et al., 2010; Banerjee and Roychoudhury, 2015). Various WRKY transcription factors have been found to be involved in ABA signaling. WRKY18 and WRKY60 act as positive regulators of ABA signaling during seed germination, and stress response while WRKY40 has the opposite effect on ABA signaling. WRKY18 and WRKY60 act as weak transcriptional activator whereas WRKY40 binds to the promoters of multiple stress-inducible transcription factor genes, including *DREB1A/CBF3, DREB2A*, and *MYB2*, and represses their expression (Chen et al., 2010; Shang et al., 2010). ABA signal perception leads to induction of WRKY18 and WRKY40 and their product could bind to W-box present in WRKY60 promoter and thereby induce it (Chen et al., 2010), (**Figure 1**). In another report, WRKY gene, *WRKY63/ABO3* (*ABA Overly Sensitive3*) has been demonstrated to be involved in drought responses. *abo3* mutant exhibits hypersensitive response for ABA in the seedling stage as well as reduced drought tolerance. WRKY63 has been shown to bind to the promoter of *AREB1/ABF2* and thereby positively regulating its expression (Ren et al., 2010). Apart from these WRKY genes, many other have been reported to be involved in drought and salt stress responses (Bakshi and Oelmüller, 2014; Banerjee and Roychoudhury, 2015)

In addition to these, NF-Y (nuclear factor-Y) transcription factors also take part in drought stress response and tolerance mechanisms. NF-Y transcription factors are heterotrimeric proteins with three distinct subunits NF-YA, NF-YB, and NF-YC and bind to CCAAT box in the promoter region of target genes (**Figure 1**). NF-Y transcription factors are crucial factors in nodulation in nitrogen-fixing plants and nitrogen assimilation but there are several reports suggesting their role in stress responses especially in drought stress response and tolerance (Li et al., 2008; Petroni et al., 2012; Laloum et al., 2013; Xu et al., 2014; Quach et al., 2015). Under drought stress, *AtNF-YA5* has been shown to be upregulated in ABA-dependent manner in leaf and roots of *Arabidopsis* plants (Li et al., 2008), (**Figure 1**). Transgenic plants overexpressing *AtNF-YA5* exhibited improved drought resistance and reduced water loss; whereas, *Atnf-ya5* mutant plants were found to be hypersensitive to drought (Li et al., 2008).

## Interaction Between Different Transcription Factors Involved in Drought Stress Response

Evidence suggests that drought-responsive transcription factors work cooperatively to regulate gene expression. The subclass III SnRK2s play a very important role in this context. These subclass III SnRK2s are induced by both ABA and drought stress. In-gel kinase assay using ABA-insensitive and –deficient mutants has suggested that drought stress activate SnRK2s independent of ABA (Yoshida et al., 2006; Boudsocq et al., 2007), (**Figure 1**). Transcriptome analysis of *srk2d/e/i* triple mutants suggests that they modulate the expression of genes involved in ABA-dependent as well as ABA-independent pathways (Fujita et al., 2009). Recently, phosphoproteome study has shown that 5 min of ABA treatment results in phosphorylation of phosphopeptide corresponding subclass III SnRK2s, but osmotic stress fails to do the same. However, short osmotic stress led to phosphorylation of a phosphopeptide corresponding to subclass I SnRK2s, which are not activated by ABA, and phosphopeptides corresponding to MAP3K and MAP4K (E Stecker et al., 2014). Novel proteins are thought to be involved in osmotic stress-dependent activation of SnRK2s.

As mentioned earlier, the target site of DREB/CBF, i.e., DRE/CRT motif also function as a coupling element for ABRE and is present in many ABA-inducible drought responsive genes (Narusaka et al., 2003). AREB/ABF proteins have been shown to physically interact with DREB/CBFs including DREB1A, DREB2A, and DREB2C (Lee et al., 2010), (**Figure 1**). Recent reports have shown that ABRE sequence in the promoter region is required for induction of *DREB2A* under osmotic stress. Furthermore, transient-expression analyses coupled with ChIP (Chromatin Immunoprecipitation) assays has shown AREB/ABFs such as AREB1, AREB2, and ABF3 can bind to the promoter of DREB2A and thereby induce them in an ABRE-dependent manner (Kim et al., 2011), (**Figure 1**). In *grf7* mutant expression of ABA-inducible genes and osmotic stress-responsive genes is upregulated (Kim et al., 2012a). All these reports suggest a complex interaction between the AREB and DREB regulons that need to be further studied in order to create a more comprehensive picture of transcriptional regulation under drought stress.

There are several reports indicating the interaction between AREB/ABFs and NACs. SNAC transcription factor *ATAF1* has been reported to bind to the promoter of NCED3 and thereby regulating ABA hormone levels, giving rise to a probability those SNACs may be involved in regulation of ABA-dependent gene expression of AREB/ABF regulons (Jensen et al., 2013). Conversely, ABRE sequences have been reported in the promoter region of SNAC genes (Nakashima et al., 2012). ANAC096 directly interacts with ABF2 and ABF4 but not with ABF3. Evidence suggests that ANAC096 acts cooperatively with ABFs in activation of ABA-inducible drought responsive genes (Xu et al., 2013), (**Figure 1**). Sakuraba et al. (2015) found that ANAC016 negatively regulates drought stress tolerance. They also found that ANAC016 directly binds to the promoter of *AREB1* and represses its expression. In addition to these two, five other drought-responsive NAC transcription factors (NAC019, NAP, NAC053/NTL4, NAC055, and NAC072) have been found to be associated with ABA signaling (Tran et al., 2004; Lee and Park, 2012; Zhang and Gan, 2012); suggesting they might act as additional tier in regulation of ABA-dependent drought responsive genes.

## Conclusion

Plant response to drought is a complex process comprising many changes from morphological to molecular level. Under drought stress, many transcription factors operate both exclusively and cooperatively forming a web of interactions. In this review, we have summarized major transcription factors that play a pivotal role in drought stress response and tolerance.

Drought activates many pathways in plants that have been broadly classified in two categories, i.e., ABA-dependent pathways and ABA-independent pathways. AREB/ABFs, DREBs and NACs are the vital transcription factors regulating a large fraction of drought inducible genes (**Figure 1**). Along with these, some other transcription factors such as MYB/MYC factors, WRKY and NF-Y have also been demonstrated to be involved in one or more drought responsive mechanisms (Abe et al., 1997, 2003; Yamaguchi-Shinozaki and Shinozaki, 2006; Baldoni et al., 2015). Four AREB/ABFs, AREB1/ABF2, AREB2/ABF4, ABF3, and ABF1 are the central players of ABA-mediated regulation of gene expression. These AREB/ABFs carry out ABA-regulated responses through binding to the ABRE *cis*-elements present in the promoter region of target genes (Yoshida et al., 2015). On the other hand, DREB1/CBF and DREB2 transcription factors regulate gene expression in ABA-independent manner. Three DREB1/CBFs, DREB1A/CBF3, DREB1B/CBF1, and DREB1C/CBF2 regulate cold-responsive gene expression whereas, DREB2A and DREB2B are mainly involved in regulation of osmotic stress-responsive gene expression (Nakashima et al., 2009). Additionally, heat stress-inducible genes are also regulated by DREB2A (Mizoi et al., 2012). However, under unstressed conditions, DREB2A levels are tightly regulated at the transcriptional and post-transcriptional levels through GRF7 and DRIPs, respectively (Yoshida et al., 2014). These DREBs bind to DRE/CRT element present in the promoters of genes acting downstream to them (Nakashima et al., 2009). Different NAC factors regulate drought-inducible gene expression by binding to NACRS *cis* elements (Nakashima et al., 2012, 2014).

Drought-responsive transcription factors interact with each other as well as components of other stress pathways resulting in overlap of target genes of these pathways. In this context, subclass III SnRK2s might act as a nodal point as these kinases can regulate the expression of AREB/ABFs as well as DREB2A (Yoshida et al., 2014). Furthermore, there are several pieces of evidence suggesting that AREB/ABF transcription factors interact with DREB and NAC transcription factors (Nakashima et al., 2014).

Transcription factors are potent candidates for engineering stress tolerant plants as a single transcription factor can modulate a large set of genes. Many of drought-responsive transcription factors have been used to improve drought tolerance in different crops such as rice, wheat, soybean, and maize (Nakashima et al., 2014; Krannich et al., 2015).

All these transcription factors along with other interacting partners constitute a complex network that has been extensively studied but not yet completely understood. In future, further studies about transcriptional regulatory network should provide a more comprehensive picture of the pathways as well as cross-talks. Identification of crucial factors of these pathways along with evolution of new technologies to produce genetically engineered plants should lead to development of plants with improved drought tolerance under field condition with minimal negative effects on crop yield and development.

## Conflict of Interest Statement

The authors declare that the research was conducted in the absence of any commercial or financial relationships that could be construed as a potential conflict of interest.
